# Genistein suppresses the proliferation of telomerase‐negative cells

**DOI:** 10.1002/fsn3.382

**Published:** 2016-05-26

**Authors:** Chuan‐Chuan Lin, Meng‐Hsun Hsieh, Shu‐Chun Teng

**Affiliations:** ^1^ Department of Food ScienceChina University of Science and TechnologyTaipei115Taiwan; ^2^ Department of MicrobiologyCollege of MedicineNational Taiwan UniversityTaipei100Taiwan

**Keywords:** Cancer, genistein, isoflavones, recombination, telomere, topoisomerase II

## Abstract

In both tumor and yeast cells that lack telomerase, telomeres are maintained via an alternative recombination mechanism. In this study, we tested genistein, a potential TOP2 inhibitor required for telomere–telomere recombination, on the repression of telomere–telomere recombination. Genistein on the repression of type II recombination on a *tlc1* yeast strain was examined by the telomeric DNA structures using Southern blot analysis. Telomere patterns of freshly dissected *tlc1* spores containing an empty plasmid (pYES2) or a yeast *TOP2* (yTOP2) plasmid were analyzed. The results indicated that the reintroduction of *TOP2* recovered the type II pattern, implying genistein in the blockage of type II survivors in the *tlc1* strain. The effects of genistein on both *tlc*1 and *tlc1 rad 51* strains in liquid and solid mediums were also examined. Finally, treatment of 10 *μ*mol/L of genistein showed inhibitory effect on the growth of telomerase‐negative U2OS alternative lengthening of telomere (ALT) cells, but not in telomerase‐positive HCT116 cells. These results provide evidences that the inhibitory effects of genistein on telomerase‐negative cells depend on type II recombination pathway in yeast and the ALT pathway in human tumors.

## Introduction

Telomeres, the protein–DNA structures found at the natural ends of eukaryotic chromosomes, are required to protect chromosomes from degradation and end‐to‐end fusion and to facilitate their complete replication. In most organisms, telomeric DNA consists of a short, tandem‐repeated sequence that has clusters of G residues in the strand that runs 5′ to 3′ toward the chromosome end and is protected by several binding proteins (Zakian 1996). It was first proposed by Watson (1972) that the DNA polymerases were unable to completely replicate the terminal lagging single strand DNA segment. Without a special mechanism, telomeric DNA would become shorter with every cell division. In most eukaryotes, telomere maintenance is carried out by a special reverse transcriptase named telomerase, which uses a small CA‐rich stretch in its RNA component to template extension of the G‐rich strand (Zakian 1996; Blackburn 1992). When any of the genes that are essential for the telomerase pathway is deleted, telomere length gradually shortens, chromosome loss increases, and most cells die (Lundblad and Szostak 1989).

Even in organisms that normally rely on telomerase, coexisting telomerase‐independent mechanisms allows cells to pass through crisis. The generation of survivors in the absence of telomerase has been studied most extensively in *Saccharomyces cerevisiae* (Lundblad and Blackburn 1993). In this pioneering study, the authors described two types of telomerase‐independent survivors, type I and type II. The majority of cells that survive in the absence of telomerase activity contain multiple tandem copies of the subtelomeric Y’ element and very short terminal tracts (Teng and Zakian 1999) (type I survivors). In a minor fraction of the survivors (type II), the lengths of the telomere sequences are increased heterogeneously from several hundred base pairs to 10 kb or longer (Teng and Zakian 1999). The generation of type II survivors is dependent on the presence of Rad50, Rad59, Rap1, Sgs1, Top3, and Top2 (Chen et al. 2001; Huang et al. 2001; Johnson et al. 2001; Teng et al. 2000; Tsai et al. 2006). The structure of type II telomeres in *Saccharomyces* resembles that of 15% of human cell lines and tumors that maintain telomeric DNA via the alternative lengthening of telomere (ALT) pathway as in some cancer cells to replicate their telomeres by telomere–telomere recombination (Bryan et al. 1997; Dunham et al. 2000; Reddel et al. 2001).

Telomerase inhibitors were discovered right after the cloning of yeast and human telomerase (Lingner et al. 1997). Telomeres are a rational target for anticancer therapeutics (Buseman et al. 2012). To date, inhibitors that modulate telomere replication have only been described for telomerase‐positive cells. Unfortunately, experiments have demonstrated that after treating telomerase inhibitors, tumor cells could switch to the ALT pathway to maintain their telomeres. The alternative ALT pathway may lead to therapeutic failures and/or acquired resistance during telomerase inhibition‐based anticancer therapy (Henson et al. 2002; Bechter et al. 2004; Hu et al. 2012; Shay et al. 2012). Therefore, the only way to make this anti‐cancer approach to work by disrupting unlimited telomere maintenance is to use cocktail drugs that contain both telomerase and ALT inhibitors (Shay et al. 2012).

Isoflavones are phytochemicals that often occur in the plant family of Leguminosae. Hundreds of studies have reported the antitumor activities of isoflavones in its mechanism of action in normal and malignant human and animal cells, animal models, in vitro experiments, or phase I/II clinical trials (Magee et al. 2004; Cornwell et al. 2004). In addition to their actions as partial estrogen agonists or antagonists, genistein (4sym, 5, 7‐trihydroxyisoflavone), the most well‐studied isoflavone in the literatures, has been shown to inhibit protein tyrosine kinase and topoisomerase I and II (Akiyama et al. 1987; Markovits et al. 1989; Boege et al. 1996). Topoisomerase are essential enzymes in cell proliferation in all living organisms since they are involved in DNA processes such as replication, transcription, translation, recombination, and chromosome dynamics, simply by regulating DNA topology. Type I topoisomerase is a monomeric enzyme that breaks one DNA strand to permit another DNA to get in (Jaxel et al. 1991). Type II topoisomerase is a dimeric and ATP‐dependent enzyme that breaks both DNA strands at once, allowing the entry of another intact DNA helix (Heck and Earnshaw 1986).

Our previous study on the effect of isoflavones on E2‐ER‐ERE‐dependent pathway indicated that the mechanism of the anti‐cancer activity of isoflavones is complicated and other mechanisms might be involved (Lin et al. 2008). As mentioned above, in order to investigate the molecular mechanism of telomere–telomere recombination, we have identified factors required for this pathway (Teng and Zakian 1999; Teng et al. 2002; Tsai et al. 2002). But so far all those 15 factors discovered by us are either essential genes or genes which encode proteins that have difficulties of developing drugs to block their activities. DNA topoisomerase displays relaxing activity on supercoiled DNA and is required for several steps during DNA metabolisms including DNA replication, recombination, RNA transcription, and chromosome segregation (Kim and Wang 1992). In our recent findings, we demonstrate that TOP2 and TOP3a are required for telomere–telomere recombination in yeast and in ALT‐type cancers (Tsai et al. 2006; Hsieh et al. 2015). Here we showed that an isoflavone and potential topoisomerase inhibitor, genistein, prevents telomere recombination in yeast and suppresses cell proliferation in ALT‐type cancers.

## Materials and Methods

### Yeast strain and culture condition, DNA preparation, enzyme digestion, gel electrophoresis, and Southern blot analysis

All the yeast operations were performed by standard methods. Yeast strains used in this study were the derivatives of YPH501 (*MAT**a**/MATα ura3‐52/ura3‐52 lys2‐801 amber/lys2‐801 amber ade2‐101 ochre/ade2‐101 ochre trp1Δ63/trp1Δ 63 his3Δ 200/his3Δ200 leu2‐Δ1/leu2‐Δ1*). The yeast strains carrying *tlc1* were described previously (Teng and Zakian 1999; Teng et al. 2000; Tsai et al. 2002). The *TOP2*::*kan* ^R^ disruption removes amino acids 6–698 of the 702 amino acid *TOP2* open reading frame by the *URA3* marker. These heterozygous diploids were then sporulated and screened for the *URA3* and *LEU2* markers by replica plating to selective medium. The pRS314y*TOP2* plasmid was constructed by cloning the PCR‐amplified 3.3‐kb EcoRI‐EcoRI fragment of the yeast *TOP2* gene into the vector.

The *rad51::HIS3* mutation was precise deletion of the *RAD51* made by transforming cells with a PCR‐generated fragment using *HIS3* as a template and primers for sequences flanking *RAD51*. Cells were serially diluted into or restreaked onto YEPD medium. To isolate individual survivors, spore clones were streaked on plates and allowed to grow for 3 days at 30°C prior to restreaking. When senescence was observed, tiny colonies were streaked and allowed to grow for 6 days. Survivors, identified by their large size in the background of slow growing, senescing colonies, were inoculated into liquid medium and grown for 2 days at 30°C for DNA preparation. Liquid survivors were isolated by inoculating spore colonies from the tetrad plate into 10 mL liquid medium. Cultures were diluted repeatedly 1:10,000 into fresh medium at 48 or 72 h intervals. When DNA was examined from individual colonies, the colony was expanded in 2 mL of liquid medium to obtain enough DNA for Southern analysis. To determine the fraction of type I and type II survivors in the different genistein‐treated backgrounds, approximately 80–100 survivors were isolated from each of two independent genistein‐treated *tlc1* spore clones. As reported previously, *rad51 tlc1* cells senesced extremely rapidly, with senescent cells evident in the first restreak. Survivors were relatively rare in the *rad51 tlc1* strain compared to their abundance in other strains. Each spore colony was inoculated into YEPD and grown at 30°C. Several independent isolates for each genotype were analyzed. Genomic DNA preparation and Southern blot analysis were performed as previously described (Tsai et al. 2002). To examine the DNA from individual colony, each colony was expanded in 2 mL of liquid medium to obtain DNA for Southern blot analysis. The DNA was digested with XhoI in order to observe type I pattern or with a mixture of HaeIII, HinfI, Hinp1I, and MspI four base cutters to observe type II pattern. The following probes were used in Southern hybridization: a 270‐bp C_1‐3_A fragment, and probes were randomly labeled with the random prime labeling system (Invitrogen).

### Growth curves of genistein‐treated *tlc1* and *tlc1 rad51* in liquid medium

Two independent spores of each genotype were used to inoculate cultures. Cultures were inoculated directly from the tetrad master plate into liquid YEPD medium and allowed to grow on 30°C roller drum until the culture is saturated. The cell concentration in each culture was determined by hemacytometer, and each culture was diluted to a concentration of 2 × 10^5^ cells/mL in fresh medium. The growth curves of genistein‐treated tlc1 and *tlc1 rad51* strains were determined for several days. The concentrations of the three cultures for each strain on each day were then averaged, and the results of the growth curves were graphed.

### Mammalian cell culture and MTS assay

Cells were cultured in Dulbecco's modified Eagle's medium (U2OS, HCT116) media containing fetal calf serum, penicillin, streptomycin, glutamine, nonessential amino acids (Hyclone). The long‐term growth curves of both 10 *μ*mol/L genistein‐treated U2OS and HCT116 cells were then determined by MTS assay. The experiment was conducted for 60 days with measuring the population doublings (PD) every 2–4 days after each dilution. PD 0 is defined as the point at which the pooled selected population was subcloned by limited dilution.

## Results and Discussion

### Effect of genistein on the type II‐surviving *tlc1* strains

**Figure 1:**
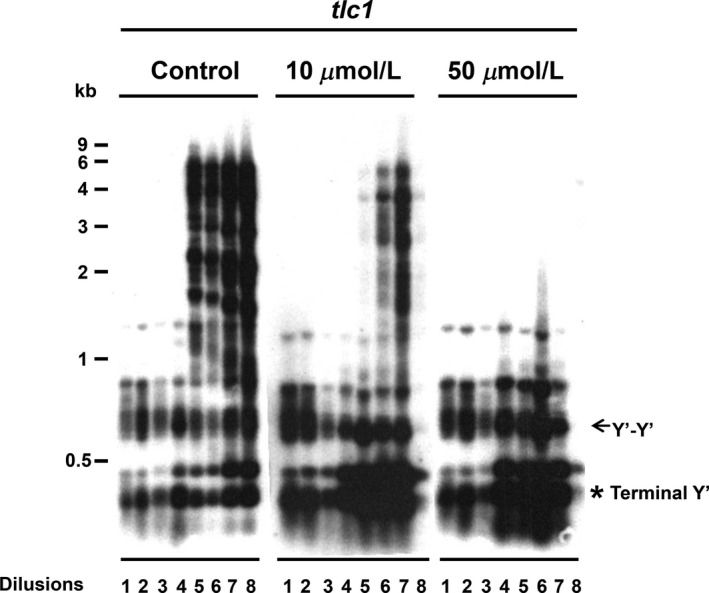
The telomere pattern of *tlc1* after genistein treatment. Southern blot of four base cutters digested genomic DNA isolated from serial liquid dilutions of *tlc1* (the telomerase RNA gene in yeast) and genistein‐treated *tlc1* strains with a telomere probed to detect telomeric restriction fragments (TRFs).

In the *tlc1* strain that lacks telomerase activity, yeast cells gradually lose viability after 40–60 generations of propagation. However, a subset of cells in the culture eventually bypass the senescence and survive. Two classes of survivors, type I and type II, have been identified and can be distinguished by two different Southern blot analyses (Teng and Zakian 1999). Type II telomeric DNA digested with these enzymes yields many differently sized fragments of up to 10 kb or even larger (Fig. 1). Previous study demonstrated that Top2 is involved in telomere recombination pathway. Since isoflavones can inhibit Top2 activity, we then test the isoflavones on the repression of type II recombination. The telomere lengths in *tlc1* and isoflavones‐treated *tlc1* strains were assessed by the Southern blot analysis. The differences in telomeric DNA structure in the *tlc1* and type II survivor are easily visualized when genomic DNA is digested with a mixture of four‐base cutter (Teng and Zakian 1999). These enzymes cut frequently in complex sequence DNA, reducing most genomic DNA to very small fragments, whereas C_1‐_3A/TG_1‐3_ DNA has no sites for any of these enzymes. Freshly dissected *tlc 1* spores were inoculated in YEPD medium and grown to stationary phase. At 48‐h intervals, DNA was isolated and cultures then diluted 1:10,000 into fresh medium. This process was repeated seven times. The DNA from each of the eight time points was analyzed as shown in Figure 1. Telomeres suddenly and dramatically lengthened at a specific reproducible time point, between the fourth and fifth dilutions, as identified by type II telomeres. The type II telomere recombination was totally abolished at 50 *μ*mol/L of genistein. These data suggest that genistein inhibits type II telomere–telomere recombination.

A previous published study indicated that genistein represses telomerase activity via both transcriptional and posttranslational mechanisms in human prostate cancer cells (Jagadeesh et al. 2006). Therefore, genistein may serve as a good therapeutic candidate to abolish telomere maintenance, both telomerase dependently and telomerase independently, by the dual effects.

### Effect of genistein on *TOP2*‐overexpressing *tlc1* strains

**Figure 2:**
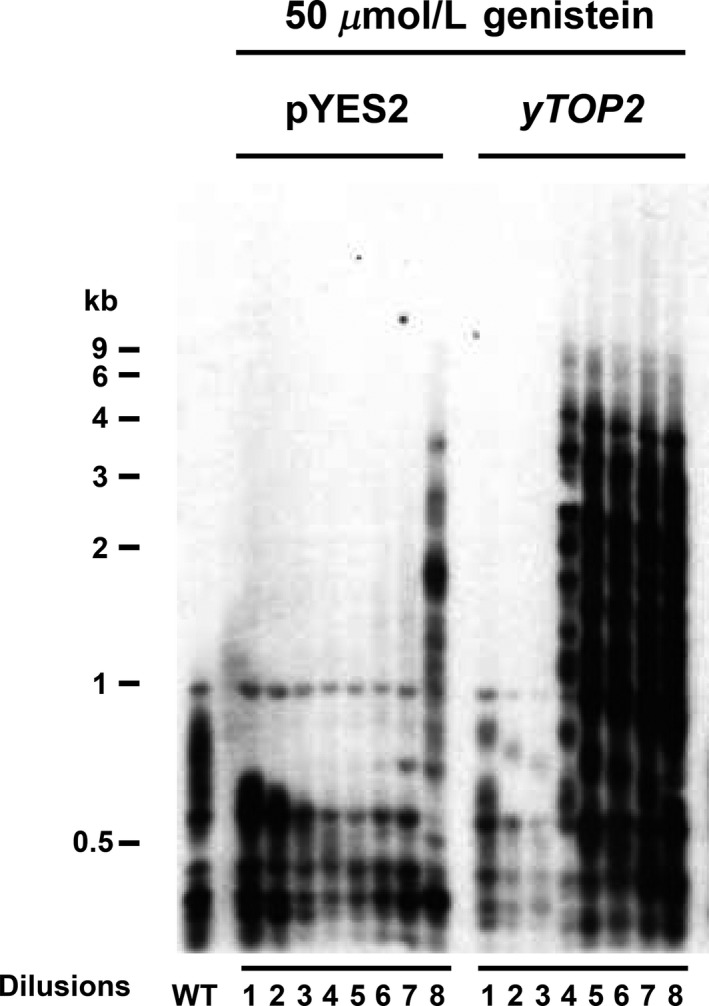
The telomere pattern of pYES2 and *yTOP2* after 50 *μ*mol/L of genistein treatment. Southern blot of four base cutters digested genomic DNA isolated from serial liquid dilutions of *tlc1* strains containing an empty plasmid (pYES2) or a yeast *TOP2* (yTOP2) plasmid treated with genistein with a telomere probed to detect telomeric restriction fragments (TRFs).

Genistein can inhibit a wide range of cancer cells, and the mechanism of genistein cytotoxicity is considered to involve in an inhibitory effect on TOP2 (Schmidt et al. 2008).We further confirmed the role that genistein abolished the generation of type II survivors specifically due to the inhibition of *TOP2*, by reintroducing *TOP2* on a low‐copy plasmid in *tlc1* strains treated with 50 *μ*mol/L of genistein. Telomere patterns of freshly dissected *tlc1* spores containing an empty plasmid (pYES2) or a yeast *TOP2* (yTOP2) plasmid were analyzed. Type II telomere pattern was recovered by reintroducing *TOP2* (Fig. 2), further suggesting that genistein abolished type II survivors through Top2 inhibition.

### Effect of genistein on *tlc1* and *tlc1 rad 51* in both liquid and solid medium

In a *tlc1*‐mutant liquid culture, although approximately 90% of the survivors are type I, they grow slowly and are quickly overtaken by the type II survivors, which have a growth rate similar to that of wild‐type cells. This is also exacerbately by the ability to gradually convert from the type I to the type II pattern of telomere structure. In the liquid culture assay, when cultures starting from freshly dissected spores were repeatedly diluted 1:10,000 at 48‐h interval, dramatic telomere lengthening could be observed after several dilution. In this study, we used this liquid culture system for all mutant strains and then conducted four‐based cutter analysis.

**Figure 3:**
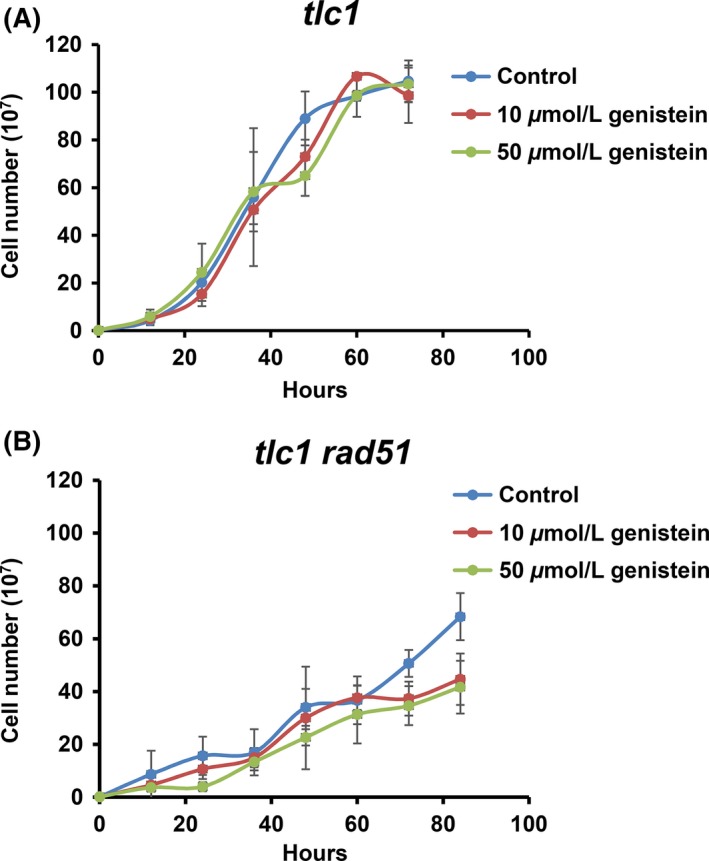
The growth curves of (A) *tlc1* and (B) *tlc1 rad51* strains under different concentrations of genistein‐treated medium. Two independent spores of each genotype were used to inoculate cultures. Each culture was diluted to a concentration of 2 × 10^5^ cells/mL in fresh medium. The growth curves of genistein‐treated *tlc1* and *tlc1 rad51* strains were determined for 72–84 h.

We first tested the effect on the growth rate of genistein‐treated *tlc1* and *tlc1 rad 51* in liquid medium. The growth curves of genistein‐treated *tlc1* and *tlc1 rad51* strains were determined for several days. The concentrations of the three cultures for each strain on each day were then averaged, and the results of the growth curves were graphed in Figure 3. In the case of *tlc1*, there is no significant difference in the growth curves of tlc1 among the control, and 10 *μ*mol/L and 50 *μ*mol/L genistein‐treated conditions. The *tlc1* growth was not affected by genistein in 50 *μ*mol/L of high concentration. In the of *tlc1 rad 51* strain, the growth rate is slower than that of *tlc1*, as shown in the recorded cell concentration every 12 h in the control. It is noticeable that significant reduction in growth was observed in the genistein‐treated *tlc1 rad51* strain after 3 days of growth. However, the reduced growth was not due to the toxicity of genistein, as proven previous in *tlc1* strain. This result suggests that the inhibitory effect of genistein is in the type II survivors’ propagation.

Top2 is important for generating type II survivors and genistein, a Top 2 inhibitor, abolishes the type II survivors. To further examine this hypothesis, we took advantage of the solid‐plate analysis. On the solid‐plate analysis, we isolated otherwise isogenic *tlc1* and *tlc1 rad51* spore products by dissecting heterozygous diploids and restreaking them multiple times on plates with genistein‐treated medium until the survivors appeared. DNA was prepared from individual survivors, and the telomere pattern was determined by Southern blot analysis. Significantly, the majority of genistein‐treated *tlc1* survivor (>97%) displayed the type I pattern, whereas both type I and type II survivors were recovered from a *tlc1* strain with a ratio of 93%:7% (Table 1). In this study, *tlc1 rad51* spores were used as a control, which exhibited exclusively type II survivors. These data suggest that type II recombination is blocked in genistein‐treated *tlc1* cells.

**Table 1:** Distribution of survivors types in *tlc1* under genistein‐treated conditions

Strain and treatment	No. of survivors (% of total)		
	Total studied	Type I	Type II
*tlc1*	92	86 (93)	6 (7)
*tlc1 rad51*	80	0 (0)	80 (100)
*tlc1* (10 *μ*mol/L genistein)	86	83 (97)	3 (3)
*tlc1* (50 *μ*mol/L genistein)	83	82 (99)	1 (1)

**Figure 4:**
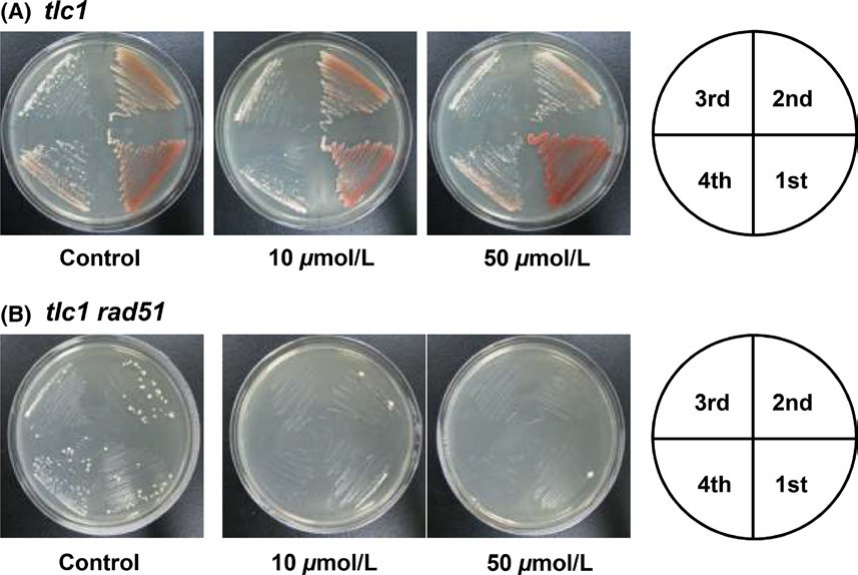
The generation growth of (A) *tlc1* and (B) *tlc1 rad51* in genistein‐treated solid plates. The diploid strain YPH501 *tlc1/TLC1 rad51/RAD51* was sporulated and dissected, and freshly isolated spore colonies were restreaked for single colonies. Each strain was repeatedly streaked on genistein‐treated solid YEPD plates and grown for 3 days at 30°C.

Cellular senescence was defined as a progressive reduction in the growth rate and an increase in the frequency of cell death in telomerase minus yeast (Lundblad and Szostak 1989). Since Rad51 is essential for type I recombination (Chen et al. 2001; Teng et al. 2000), to test whether *tlc1*,* rad51*, and genistein treatment could abolish all three telomere maintenance pathways (telomerase, type I, and type II) and accelerate cell death, strains generated from freshly dissected spores with different mutation backgrounds were examined using serial single colony restreaking on solid plates (Fig. 4). To further test whether genistein abolishes type II recombination pathway exclusively, strains generated from freshly dissected spores with *tlc1* and *tlc1 rad51* mutation backgrounds were examined by serial single colony restreaking on solid plates with different concentrations of genistein. We found that in sharp contrast to control *tlc1 rad 51* strain or genistein‐treated *tlc1* strain, which showed a cellular senescence phenotype on streaking plates, the *tlc1 rad51* spores treated with genistein could be characterized by microcolony formation from the tetrad dissections and completely lost their viabilities at the second restreak (Fig. 4). These results suggest that genistein completely block the generation of type II survivors, which causes accelerated cellular senescence.

### Effect of genistein on telomerase‐positive and ALT cells

**Figure 5:**
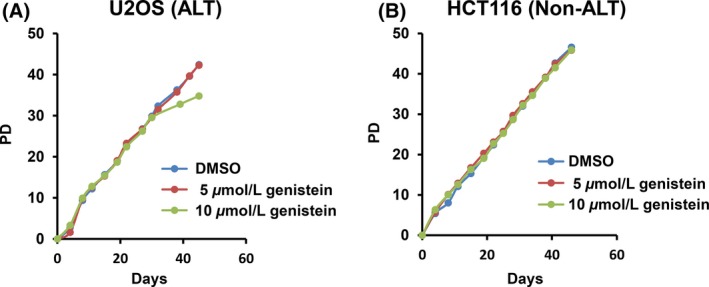
The long‐term growth curves of 10 *μ*mol/L genistein‐treated (A) U2OS and (B) HCT116 cells. The cells were equally seeded in genistein‐containing medium, and long‐term growth curves for ALT (U2OS) and telomerase‐positive cells (HCT116) were calculated.

ALT cells have very long and heterogeneous telomeres that are reminiscent of yeast type II telomeres (Bryan et al. 1995). A recent study speculated that loss of chromatin remodeler ATRX increases stalled replication forks at telomeric sites and leads to the initiation of aberrant telomeric recombination and ALT pathway activation. In addition, another study showed that loss of the histone chaperone ASF1 leads to the acquisition of several ALT phenotypes. Evidence suggests that ALT is also mediated by epigenetic regulation and gene conversion (Dunham et al. 2000; Lovejoy et al. 2012; O'Sullivan et al. 2014). The human homolog of RecQ helicase, WRN, was shown to work at the telomeres of ALT cells (Opresko et al. 2004). These results, combined with our findings raise the possibility that human TOP2 is an ALT factor, which has also been proven in our previous study (Hsieh et al. 2015). The telomerase‐positive cells HCT116 and telomerase‐negative U2OS ALT cells were treated with 10 *μ*mol/L of genistein individually. The growth curves were determined by MTS assay. The result shown in Figure 5 indicated that low dosage of genistein has little effect on ALT cell growth, however, not any effect on telomerase‐positive cell was observed. To further examine their roles in telomere formation, their telomere lengths following population doublings were analyzed. The cell lines were maintained under selection in the media for 50 PDs and their telomere length was assessed by Southern blotting using a telomeric probe. An indication of ALT telomere is the pattern of heterogeneous sizes (Bryan et al. 1997, 1995). However, no significant change in telomere pattern in ALT and telomerase‐positive cell line was observed after long‐term treatment of genistein (data not shown).

### Concluding remarks

In this study, we have demonstrated that a potential topoisomerase inhibitor, genistein, prevents telomere recombination and suppresses cell proliferation of ALT‐type cancer cells in vitro. These results might provide a valuable clue for treating telomerase‐negative cancer cells with naturally occurring phytochemicals.

## Conflict of Interest

None declared.
